# Shifting Seasons: Long‐Term Insights Into Climate Change Effects on Bird Phenology From Ringing Data

**DOI:** 10.1002/ece3.72317

**Published:** 2025-10-10

**Authors:** Danielle L. Hinchcliffe, Patrick Tkaczynski

**Affiliations:** ^1^ School of Biological and Environmental Sciences Liverpool John Moores University Liverpool UK

**Keywords:** climate change, conservation, ecology, evolution, ornithology, phenology

## Abstract

Understanding how animal communities respond to environmental change is crucial for predicting biodiversity trends. Birds, particularly migratory species and those experiencing large‐scale declines, are sensitive to shifting climatic conditions. Environmental stressors have been linked to earlier migration timing, which can alter species abundance and disrupt ecological interactions. Long‐term population monitoring provides essential insights into species' capacity to adapt to climate change, offering a predictive framework for assessing their future viability. We analyse a 25‐year bird ringing dataset from Spurn Bird Observatory located at a notable migratory bird hotspot in the UK. We show that climate factors, especially temperature, are significantly changing and consequently impact migrant bird arrival times. We also show that different species' abundances are changing over time and make a weak but notable association between these trends with climate change. When species are analysed in isolation, it is clear there are other potential contributing factors which may explain variation in abundance at Spurn over the years—to fully understand these changes, species must be studied in an ecological context, including multi‐species analyses. We take care to control for catching effort in our analyses, as we find that this directly correlates with both abundance and diversity of species caught, which demonstrates the importance of year‐round standardised ringing coverage at UK biodiversity hotspots. As such, we suggest caution when using ringing data to make ecological interpretations. While citizen science ringing data has limitations that restrict its use for elucidating mechanisms of species‐level patterns, it remains a vital tool for informing conservation. Our study highlights the value of sustained ecological datasets in tracking these dynamics and informing conservation strategies across taxa for habitat and landscape‐level management.

## Graphical abstract

This study analyses a 25‐year bird ringing dataset from Spurn Bird Observatory to assess how climate change affects migratory bird arrival times and species abundance. While there was a weak association between climate and abundance trends, phenological timings are significantly affected. Other factors influence species variation, highlighting the complexity of ecological responses. This study underscores the value and limitations of long‐term citizen science data in informing conservation strategies and emphasises the need for consistent monitoring efforts.

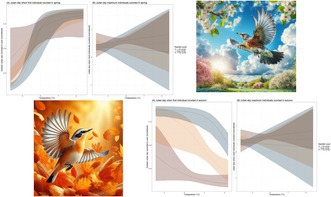

## 1 Introduction

The Earth's climate has recently changed at unprecedented rates (Wiens and Zelinka 2024). Many notable features of plant and animal populations, such as geographic distributions, life history traits and the timing of key events such as breeding and migration, are showing directional changes associated with rapid environmental change (Cleland et al. 2007; Van Buskirk et al. 2009). Studies have shown that climate (Hawkins, Field, et al. 2003; Rahbek et al. 2007) and productivity (Waide et al. 1999; Hawkins, Porter, and Diniz‐Filho 2003) determine the structure of species assemblages across large spatial scales. However, the mechanisms that drive spatiotemporal dynamics of species assemblages have received little attention so far (Jetz et al. 2005; Dornelas et al. 2013; Ferger et al. 2014).

Birds, as highly mobile and ecologically diverse organisms, are particularly sensitive to climate‐driven changes, making them valuable indicators of ecosystem health. Their shifts in migration timing, breeding success and population trends provide crucial insights into broader environmental disruptions caused by climate change. Spurn Nature Reserve has high avian biodiversity because of its unique geographic location, diverse habitats and minimal human disturbance. It is important to make use of dedicated bird observatories set up along migration flyways, as they typically obtain phenological data, either through trapping and/or by direct observation (e.g., Hüppop and Hüppop 2003). Being situated on a narrow peninsula on the east coast of England also makes it a critical stopover site for migratory birds travelling along the East Atlantic Flyway, and consequently a hotspot for both resident and migratory species.

Bird ringing datasets have been instrumental in advancing understanding of avian ecology, migration patterns and population dynamics. The large‐scale, long‐term data collected through bird ringing programmes have provided invaluable insights into bird life histories, such as migratory routes and breeding behaviours (Cox et al. 2020). For instance, studies using ringing data have revealed critical migratory stopover sites and changes in migratory timing, which are essential for conservation planning (Norris et al. 2019). However, while bird ringing datasets offer extensive longitudinal data, they also have limitations. The accuracy of the data can be affected by factors such as ringing effort, detectability biases and the potential for data loss due to non‐recovery of rings (Calvert et al. 2021). Moreover, the datasets are often skewed towards more easily captured species, potentially neglecting less accessible or rarer species (Harris et al. 2020). Despite these caveats, bird ringing remains a powerful tool for ecological research, provided its limitations are acknowledged and addressed through complementary methods and data verification techniques.

While numerous studies have documented advances in the timing of spring migration in birds over recent decades (Lehikoinen et al. 2004; Møller et al. 2008), these patterns are not uniform across species or populations. Migratory responses to climate change remain highly heterogeneous, often varying even within a species depending on location. Our study is distinctive in that it focuses on a critical yet underexplored point along the migratory route, offering rare insight into phenological shifts at a key stopover or passage location. Uniquely, we uncover a pattern of phenological change that deviates from the widely reported trend of earlier spring arrival—challenging prevailing assumptions in the literature. Understanding such location‐specific and unexpected responses is essential, as shifts in arrival timing can cascade through trophic interactions, potentially leading to mismatches between predators and peak prey availability (Visser et al. 1998; Both and Visser 2001). This underscores the need for more nuanced, geographically diverse research to inform conservation strategies at the community level.

In this study, we accessed Spurn Bird Observatory's ringing records dating from 1995 to 2020, before the covid‐19 pandemic. This viral outbreak had a significant and detrimental impact on conservation management efforts UK‐wide (e.g., the BTO Breeding Bird Survey was unable to produce population trends for 2020 due to survey restrictions) (Gillings 2022). Therefore, data between 2020 and 2024 was not yet available. We make use of this long‐term dataset to investigate species abundance and arrival dates of migratory birds, to see whether any of these metrics are influenced by (i) unprecedented acute environmental changes, and/or (ii) long‐term coastal climate change. We predict that temperature and rainfall will impact resident bird numbers as significant changes can influence breeding success and survival through affecting nesting, foraging and food availability (e.g., Dunn et al. 2010; Skagen and Adams 2012; Dunn and Winkler 2019). For migrant birds, less direct mechanisms may result in a change of arrival dates if there is a change in the stopover conditions, and there may also be a lag effect into subsequent years.

## 2 Materials and Methods

### 2.1 Data Acquisition and Availability

Standardised bird ringing data were obtained from the DeMON Database (BTO 2024a, 2024b), which stores British Trust for Ornithology (BTO) ringing records submitted by local bird ringers with Constant Effort Site (CES). This is part of a national monitoring scheme designed to track temporal and spatial changes in breeding bird populations. The CES operates under strict protocols to ensure consistency across years and locations. Birds are caught in mist nets set up in fixed positions and operated for the same number of visits and duration each year. All birds captured were ringed or identified if already ringed, and biometric data including species, age, sex, wing length and weight were recorded. While standardised BTO ringing data were available as early as 1970, only data from 1995 onwards were used in the study based on data quality checks (BTO 2024a, 2024b). Similarly, only data until 2020 were used due to the outbreak of the covid‐19 pandemic interrupting regular monitoring and data collection. Weather data were used from the publicly available HadUK‐Grid Climate Dataset, which covers the UK land area at 1 km × 1 km resolution (Met Office et al. 2021). Data on rainfall, temperature and snow fall were taken for the grids covering the Kilnsea and Spurn Point area, which is where the BTO constant effort netting sites are established for standardised bird ringing efforts. Extracted data included daily, monthly and yearly values for maximum, minimum and average measures. All data and code used in this study are stored in a repository and are available upon request.

### 2.2 Model Fitting and Validation

For our analyses, we employed a Bayesian approach with all models fitted and estimated using Hamiltonian Monte Carlo methods and Stan software (Stan Development Team 2021) with the *brms* package (Bürkner 2018) within R (R Core Team 2022).

Prior to model fitting, any potential issues of covariation or collinearity between our fixed effects were inspected via pairwise plots, pairwise correlations and variance inflation factors (VIFs). Pairwise plots and correlation coefficients were generated using the ‘covees’ function of the *GGally* package (Schloerke et al. 2024); VIFs were generated using the ‘VIF’ function of the *car* package in R (Fox and Weisberg 2019). For the phenology models, rainfall and temperature were collinear with each other but were fitted in an interaction as they could act synergistically on bird migratory behaviour. Subsequent model validations (see below) suggested no issues with this approach.

For all models, numeric variables were standardised as *z*‐scores. We fit models with weakly regularising priors for the fixed effects (*β* ~ Normal [0.1]). For the priors for the components of the random effects, we used the default priors provided by the ‘get_prior’ function of *brms*, namely a weakly regularising half student‐t prior (df = 3, scale parameter = 10) for the random intercepts and a uniform LKJ Cholesky prior (*η* = 1) for covariance matrices of the random slopes. For all models, we specified three chains of 5000 iterations, 2000 of which were devoted to the warm‐up. Sampling diagnostics (Rhat < 1.01) and trace plots confirmed chain convergence for all models. Effective sample sizes confirmed no issues with autocorrelation of sampling for all models.

To interpret the strength and uncertainty of the associations between our predictor variables and outcomes, we report the model estimate, 90% credible intervals and the proportion of posterior (*p* + or *p*−) supporting the direction (positive or negative) of the model estimate of the associations (Martin et al. 2020; McElreath 2020; McShane et al. 2019). We considered effects whose direction was supported by more than 95% of the posterior distribution to be well supported, and those whose direction was supported by more than 90% of the posterior distribution to be weakly supported. All models were validated using posterior predictive checks (see Figures S1 and S2 for plots of these checks).

### 2.3 Climate Models

To examine how the climate has changed at Spurn, we ran three models, each with ‘year’ as the numeric predictor of (a) average rainfall, (b) average temperature and (c) average snowfall (each model spanning 25 years of climatic data). Both the rainfall and temperature models were fitted with a Gaussian error structure; skew in the snowfall data meant that this model was fitted with a log‐normal error distribution.

### 2.4 Phenology Models

To examine climatic impacts on migratory patterns, we modelled the impact of a changing environment on the earliest day within spring (period defined as Julian day 60–152) and autumn (Julian day 212–304) that each species was observed within a given year. In addition, we also modelled whether changing climate was associated with changes in the Julian day when the most amount of captures for a given species occurred within each species.

For our phenology analyses, we included only species with at least 50 capture records across the entire dataset. This threshold was chosen to ensure sufficient temporal coverage over the 25‐year study period. Although over 500 species were initially available, many had records concentrated in only a short portion of the timeline, limiting their utility for long‐term trend analysis. Therefore, the final dataset analysed included 12 bird species that are migratory to Spurn at different times of the year (Table S1).

In both spring and autumn, individual species had their own range of variation in terms of arrival day and day of maximum captures. For example, in spring, redwings never arrived later than Julian day 80, whereas swifts and wheatears had the earliest arrival days across the entire range of spring. Therefore, we normalised within species the earliest arrival days and days of maximum captures so that these variables were scaled between 0 (earliest day of arrival/maximum capture for a species) and 1 (latest earliest day/maximum capture of arrival for a species) prior to modelling. As these normalised days were our response, all models were fitted using a zero–one‐inflated beta error distribution.

In all spring and autumn models, we included as a control variable the sampling effort for species within a given year, calculated as the number of times a particular species was caught divided by the overall capture rate. Our predictors were climatic variables, that is, average UK‐wide rainfall and temperature across the 90 days preceding the earliest day of arrival within each season. We did not include snowfall as only yearly rather than monthly values were available for this variable. In this analysis, we used the UK‐wide climatic predictors rather than local measures as birds will be using climatic cues from their departure site, not the arrival site, to make migratory decisions. Therefore, despite the UK having a wide variety of microclimates, we feel the UK averages have a better chance of capturing climatic trends that might predict arrival. Furthermore, all climatic recordings at Spurn were highly correlated with national averages (Figure S3 in Supporting Information), meaning the impact of using either local or national climatic variables was likely to be quite subtle.

As both temperature and rainfall may have synergistic effects on bird migratory behaviour, we included an interaction between these variables in the model. In all models, we included species as random effects (with random slopes for all predictor variables) to account for multiple observations of the same species and potential within species responses to climate; we also included a random intercept for year (as a categorical variable) to account for any within sampling year impacts not included among our fixed effects. For spring, all 12 migratory species were represented in the dataset, although not in every year; thus, the final sample size for this model was 172 spring arrival days. For autumn, all species were represented but not every year; thus, the final sample size for this model was 194 arrival days.

The models of earliest arrival times in spring and autumn were fitted using a zero‐inflated beta error distribution; the models for maximum number of arrivals in spring and autumn were fitted using a Gaussian error distribution.

### 2.5 Abundance Models

For our abundance model, annual capture rates per species adjusted for sampling effort (number of captures per species divided by overall number of captures) were the response variable. Again, only species with catch counts of 50+ within a year were included in the analysis, as well as only species that we could assign as residents and/or migratory to Spurn at different times of the year. Therefore, the final dataset analysed included 25 bird species, with 510 yearly abundance values, a mean ± SD of 20.40 ± 3.28 yearly abundance values per species.

Again, capture rates varied substantially between species, so we normalised this variable within species between 0 and 1. For our analysis, we fitted three‐way interactions between our climatic variables (yearly rainfall and temperature) and the migratory status of the species (three levels: resident, migratory, mixed status). However, we found no meaningful association between these interactions and annual abundance rates, and therefore, ran the model with only the single terms included (climatic variables and migratory status of the species). We again included species identity as a random intercept, with random slopes for our climatic variables, to account for multiple observations of the same species and potential within species responses to climate.

In addition, to examine how abundances generally changed over time, we fitted a model with year as a numeric predictor of normalised abundance.

All abundance models were fitted with a zero–one‐inflated beta error distribution.

## 3 Results

From 1995 to 2020 at Spurn Nature Reserve, temperatures tended to increase over time (*p* + = 0.938), with an estimated increase of 0.034°C per year; in the same period, yearly snowfall has clearly declined (*p* − = 0.989). There was no discernible change in rainfall (*p* + = 0.572).

### 3.1 Phenology

#### 3.1.1 Spring

**FIGURE 1:**
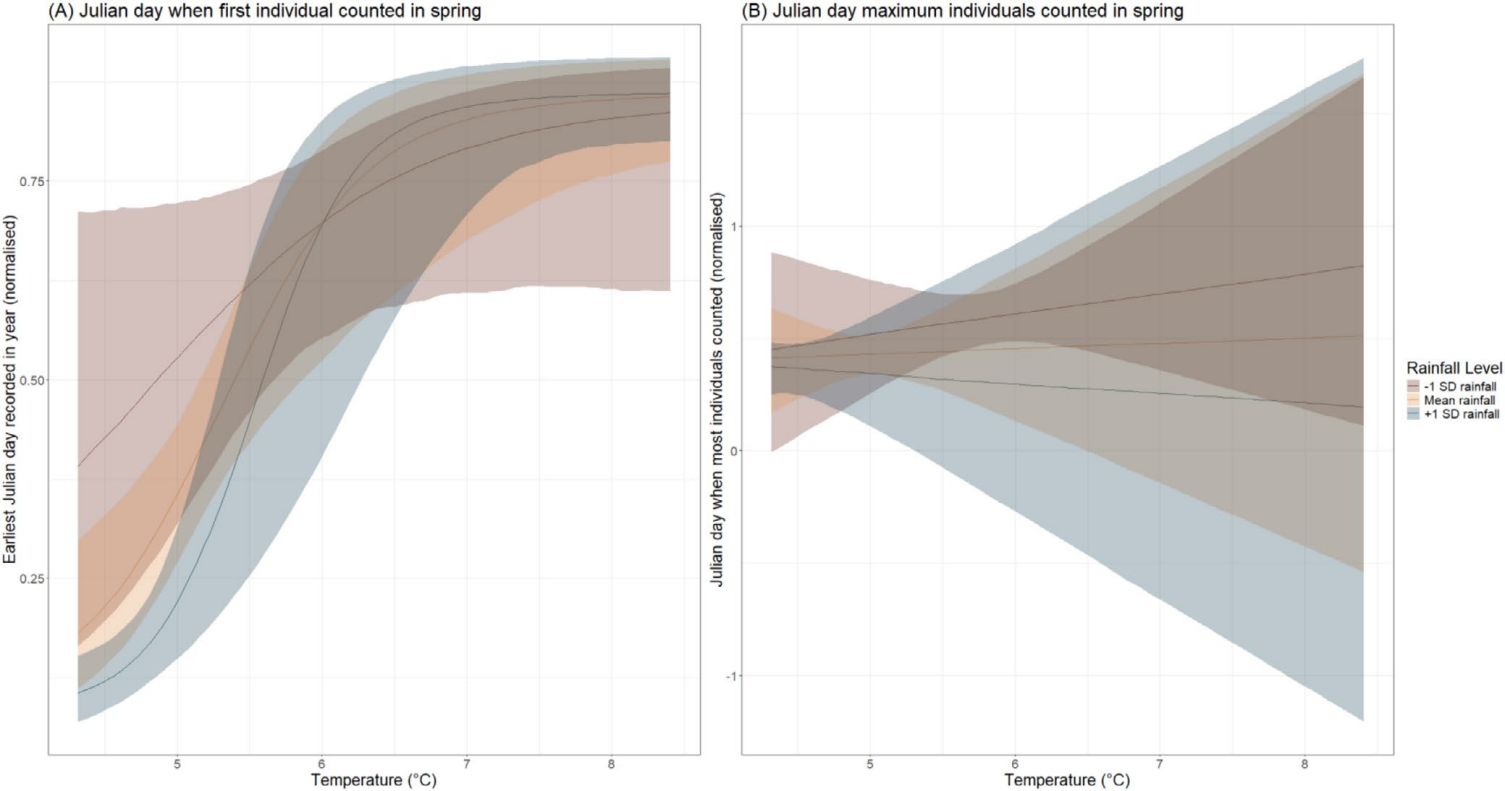
Interactive effects between average temperature (°C) and rainfall (mm) with (a) earliest arrival day within spring (normalised within each species between 0 and 1) and (b) day when maximum number of individuals were counted in spring in each species. Rainfall was fitted as a continuous variable and is only categorised for visual purposes.

There was a clear and consistent association between the interaction between average temperature and rainfall and the earliest Julian day of arrival in spring (*p* + = 1.000; Table 1; Figure 1), wherein warmer and wetter springs were associated with later arrival dates. Although the interaction between rain and temperature was also clearly associated with the Julian day when the maximum number of individuals was caught across species (*p*− = 0.956; Table 1), the effect was clearly very weak and likely not biologically meaningful. In springs with mean rainfall, temperature was not associated with the Julian day when most individuals were counted; in wetter springs, the day of maximum counts was slightly earlier in the season; when dryer, this day occurred slightly later (Figure 1).

**TABLE 1:** Model results exploring how climatic factors relate to (a) earliest arrival day in spring, (b) day of maximum number of arrivals in spring, (c) earliest day of arrival in autumn and (d) day of maximum arrival in autumn for migratory birds in Spurn. Coefficients in bold had estimate directions supported by > 95% of the posterior distribution.

Coefficient	Estimate	Error	Lower 95% CI	Upper 95% CI
**Spring Phenology: Earliest arrival**				
Intercept	−0.458	0.224	−0.916	−0.026
Temperature	1.657	0.445	0.729	2.462
Rainfall	−0.854	0.416	−1.711	−0.080
Sampling effort	−0.552	0.933	−2.356	1.276
**Temperature**: **Rainfall**	**0.786**	**0.072**	**0.646**	**0.930**
**Spring Phenology: Maximum # arrivals**				
Intercept	0.422	0.047	0.329	0.512
Temperature	0.023	0.141	−0.240	0.310
Rainfall	−0.090	0.119	−0.317	0.151
Sampling effort	0.904	0.916	−0.883	2.682
**Temperature: Rainfall**	**−0.058**	**0.034**	**−0.125**	**0.009**
**Autumn Phenology: Earliest arrival**				
Intercept	−0.231	0.246	−0.727	0.238
**Temperature**	**−1.14**	**0.401**	**−1.865**	**−0.279**
**Rainfall**	**2.395**	**0.392**	**1.526**	**3.073**
Sampling effort	−0.215	0.99	−2.162	1.696
**Temperature: Rainfall**	**−0.407**	**0.099**	**−0.608**	**−0.221**
**Autumn Phenology: Maximum # arrivals**				
Intercept	0.42	0.047	0.328	0.514
Temperature	0.018	0.143	−0.258	0.308
Rainfall	−0.093	0.120	−0.322	0.155
Sampling effort	0.904	0.893	−0.863	2.678
**Temperature: Rainfall**	**−0.06**	**0.035**	**−0.132**	**0.009**

#### 3.1.2 Autumn

**FIGURE 2:**
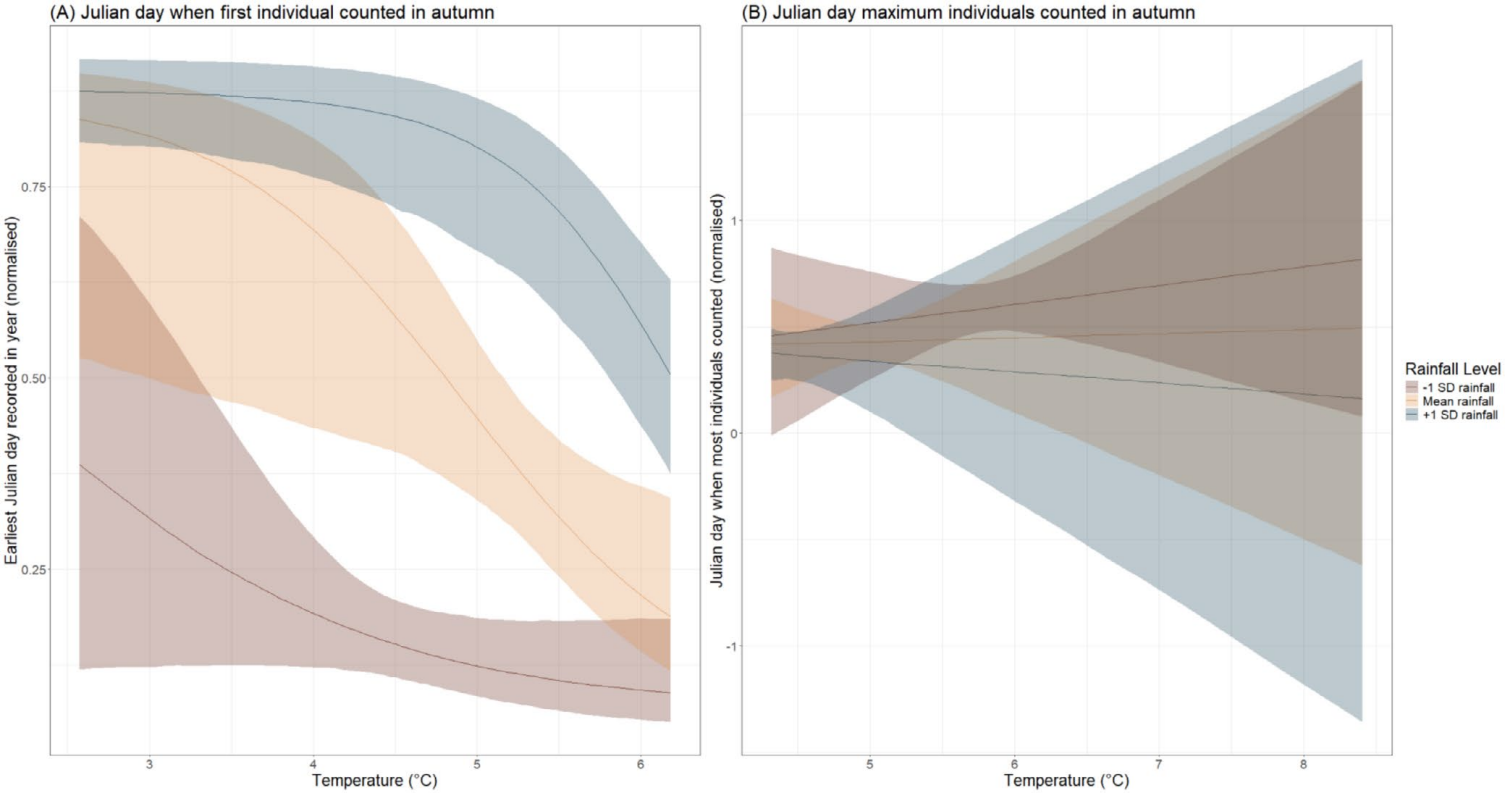
Interactive effects between the interaction between average temperature (°C) and rainfall (mm) with (a) earliest arrival day within autumn (normalised within each species between 0 and 1) and (b) day when maximum number of individuals were counted in autumn in each species. Rainfall was fitted as a continuous variable and is only categorised for visual purposes.

The interaction of average temperature and rainfall with the earliest Julian day of arrival in autumn was evident and persistent (*p*− = 1.000; Table 1; Figure 2), wherein warmer and drier years tended to lead to earlier arrival dates. In wetter years, arrival dates were generally later in autumn; however, warm and wet autumns saw earlier arrival dates. As with our spring analysis, we found a clear association between the interaction of rain and temperature and the Julian day when most individuals were counted for a species; however, this association was again very small in size and likely not biologically meaningful. In drier, warmer years, the day of maximum counts tended to occur later in spring, whereas in wetter, warmer years, the day of maximum counts occurred earlier in autumn (Figure 2).

Based on these results, we also ran *post hoc* models looking at the length of residence in spring and autumn within any given year; this was calculated as the latest Julian day observed in spring/autumn minus the earliest Julian day observed in spring/autumn within each species in each year. Different bird species had different ranges of spring/autumn residencies. Therefore, we normalised the residence lengths within each species in each season.

We removed years in which species were only observed once in spring/autumn (and thus had a length of residence of 0), resulting in a final data set of 140 residence lengths within spring and 159 residence lengths within spring. The fixed and random effect variables in these models were the same as in our spring and autumn phenology models. Once again, as we were using a normalised response variable, these models were fitted with a zero–one‐inflated beta error distribution.

**FIGURE 3:**
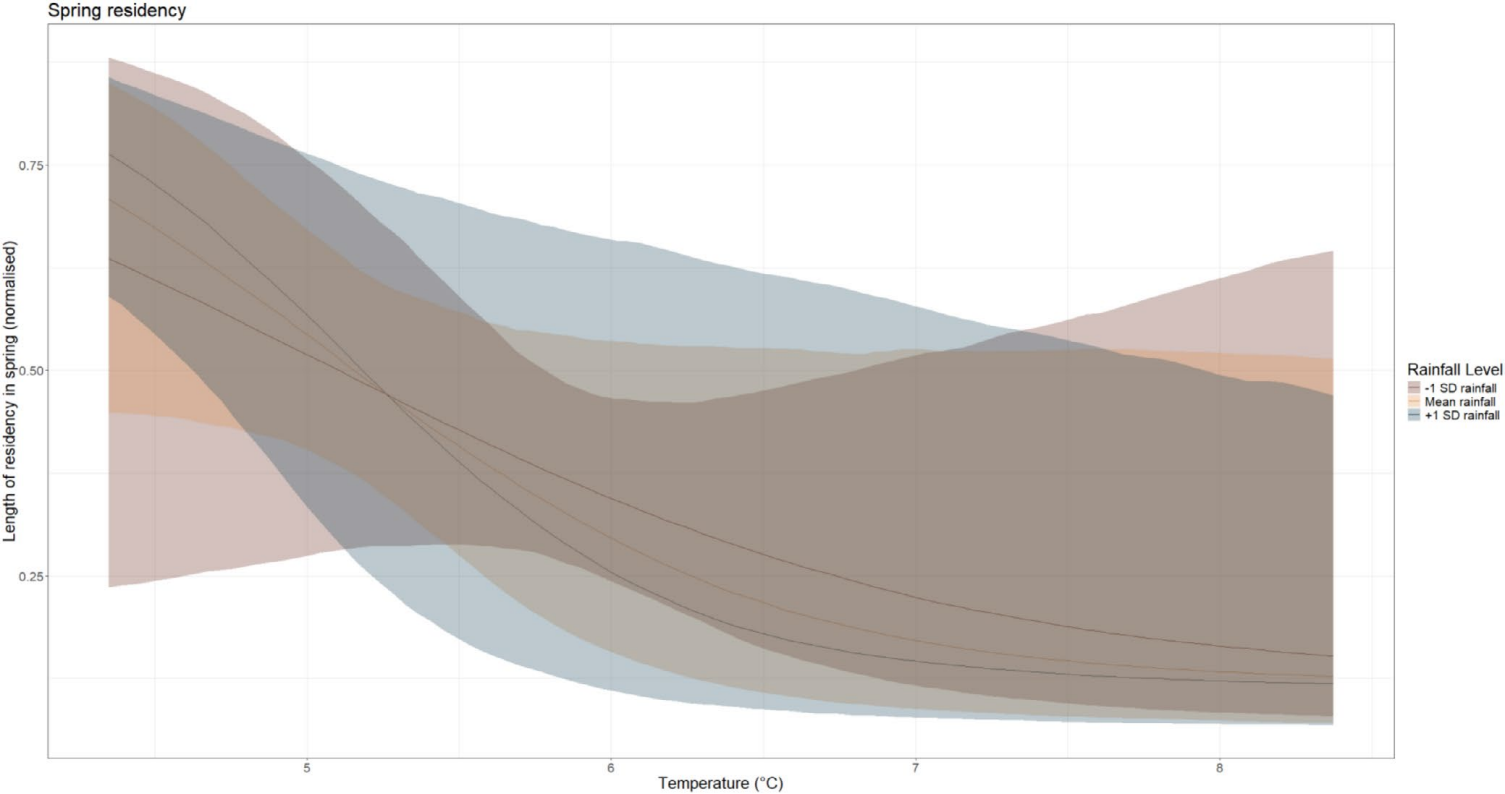
Interactive effects between the interaction between average temperature (°C) and rainfall (mm) with residency length in spring (normalised). Rainfall was fitted as a continuous variable and is only categorised for visual purposes.

There was a strong and reliable association between the interaction of average temperature and rainfall and spring residency length (*p*− = 0.998; Table 2; Figure 3). Warmer years were generally associated with shorter residencies, with warm and dry years more likely to have shorter residencies than warm and wet years. However, there was no clear association between our climatic variables and autumn residency (*p* + = 0.881; Table 2).

**TABLE 2:** Model results exploring climatic factors related to residence length in (a) spring and (b) autumn for migratory birds in Spurn. Coefficients in bold had estimate directions supported by > 95% of the posterior distribution.

Coefficient	Estimate	Error	Lower 95% CI	Upper 95% CI
Spring				
Intercept	0.028	0.317	−0.609	0.659
Temperature	−1.158	0.600	−2.347	0.017
Rainfall	0.080	0.556	−1.009	1.142
Sampling effort	0.026	0.983	−1.942	1.928
**Temperature: Rainfall**	**−0.377**	**0.132**	**−0.632**	**−0.121**
Autumn				
Intercept	0.429	0.187	0.041	0.786
Temperature	0.584	0.344	−0.119	1.256
**Rainfall**	**−1.356**	**0.367**	**−2.062**	**−0.585**
Sampling effort	0.150	0.995	−1.824	2.069
Temperature: Rainfall	0.142	0.120	−0.087	0.379

Taking the results of these models in combination, this suggests that in warmer and wetter years, birds are arriving later in spring but staying for less time. In warmer and drier autumns, birds are arriving later but not necessarily staying any longer or shorter.

### 3.2 Abundance

**FIGURE 4:**
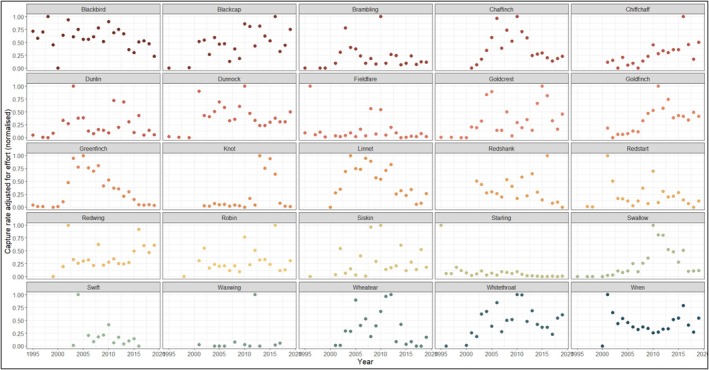
Changes in capture rate adjusted for effort for selected bird species between 1995 and 2020 at Spurn. Abundances were normalised within each species due to substantial between‐species variation in mean capture rates. Points are coloured by species to improve the readability of the plot.

Visual inspection of capture rates (normalised within species) suggested divergent patterns among species in their abundances in recent years (Figure 4). For example, greenfinches have seen a sharp decline in abundances in recent years, whereas blackcaps and chiffchaffs have largely increased in abundance in recent years. However, when we fit our model to include climatic predictors, we observed generally weak and uncertain positive associations between abundances and all our climatic predictors (Table 3a). Overall, it seemed climate was not a strong predictor of abundance in our analysis. When looking at general changes over time, there was a clear but extremely weak positive association between the year of observation and normalised abundances (Table 3b).

**TABLE 3:** Results from model exploring relationships between climatic factors and bird species abundances in Spurn. Coefficients in bold had estimate directions supported by more than 95% of the posterior distribution. For the categorical variable, the reference category is in parentheses.

Coefficient	Estimate	Error	Lower 95% CI	Upper 95% CI
Climate as predictor				
Intercept	−1.100	0.195	−1.490	−0.709
Rainfall	0.033	0.052	−0.068	0.136
Temperature	0.042	0.058	−0.071	0.157
Status (Migratory)				
Mixed	0.409	0.269	−0.125	0.920
Resident	0.629	0.569	−0.488	1.724
Year as predictor				
Intercept	−46.241	14.412	−73.960	−18.688
Status (Migratory)				
Mixed	0.482	0.272	−0.076	1.022
Resident	0.893	0.694	−0.487	2.239
**Year**	**0.022**	**0.007**	**0.009**	**0.036**

## 4 Discussion

Temperatures are clearly rising over the years at Spurn Nature Reserve, and snowfall is declining, which is consistent with broader climatic patterns across the UK and globally (IPCC 2021; Met Office 2023). While there appears to be no significant change in rainfall at this coastal reserve, the interaction between warmer temperatures and wetter seasons does play a significant role in the decision‐making of some species when it comes to staying and leaving the UK after breeding. Warmer temperatures, overall, appear to be resulting in later arrivals of birds in springtime and earlier departures in the autumn. This typically goes against findings in the wider literature. For example, one study that used a similar dataset for a site in southern England (Portland, Dorset) found 11 out of 26 species were arriving earlier in the spring, and while the monthly average temperatures along the European part of the birds' migration route were generally negatively correlated, it could only explain < 30% of the variability in first arrival dates (Croxton et al. 2006). Another study looked at the arrival and departure dates from 145 bird series across six and three UK sites, respectively and found 50% were arriving significantly earlier over the years, and just under 50% were departing later (Sparks et al. 2007). This was significant and negatively correlated to UK temperatures for 26% of all series for spring arrivals, but temperature effects on departures were less clear. The trend of earlier spring arrivals over time, in parallel with rising temperatures, has also been evidenced at larger European scales (e.g., Lehikoinen et al. 2004). However, it is important to note that response to climate change varies between areas (Sparks 1999). Differences in response arise more probably from spatiotemporal variation of climate change than from differences in birds' behaviour, but they are also not mutually exclusive. Spurn is a coastal reserve in the UK with an exceptionally diverse suite of habitats. Although it can often reflect the mainland's broader ecological patterns, it has unique characteristics that will influence bird behaviour in a multitude of ways. For example, migratory bird behaviour, which is especially true for passage birds that use the site as a migration stopover. Coastal conditions will impact how long birds stay, especially in wetter and milder years when food is more abundant, such as seeds and insects (e.g., Border et al. 2024). Coastal regions also often experience stronger, more predictable wind patterns and thermal gradients, and so migrants may time flights or adjust routes to take advantage of optimal conditions/avoid adverse conditions (e.g., Socolar et al. 2017). Furthermore, coastal‐specific mechanisms such as sea‐level rise, coastal erosion and climate‐driven storms can degrade and/or shift coastal habitats over time (e.g., Langan et al. 2021). This results in a loss of critical stopover or roosting sites and forces birds into using suboptimal habitats or spending more energy to reach inland alternatives. All of these factors interact in complex ways, meaning coastal climates can both facilitate and disrupt migration and potentially explain the patterns we have observed over the last 25 years.

It is well‐evidenced that phenological plasticity confers a fitness advantage. For example, pied flycatchers ( *Ficedula hypoleuca* ) that left African non‐breeding sites earlier in the spring resulted in greater breeding success in the UK with larger clutches and a higher rate of fledging success (Bell et al. 2024). Another study showed that blue tits ( *Cyanistes caeruleus* ) arriving later to their breeding site in spring had greater reproductive success because individuals who arrived earlier missed the peak in caterpillars, resulting from the warmer temperatures. By mistiming the peak in food abundance, chicks from the earlier broods faced a food shortage and increased mortality (Visser et al. 1998). There is some evidence that trends in arrival dates may be masked by population declines in birds (Sparks et al. 2007). As bird populations decline, the number of individuals arriving at breeding sites each year diminishes. This reduces the overall sample size available for studying arrival dates. With fewer individuals to track, it becomes harder to detect subtle trends or shifts in arrival timing. In our study, we employed Bayesian modelling, which measures uncertainty in associations, and this uncertainty will be related to the overall sample sizes included. This means that although our study is not immune to the difficulties of detecting trends when sample sizes become increasingly spare, our modelling approach is appropriate for dealing with this issue. However, statistics cannot overcome the fact that declining populations can change in distribution and in behaviour, meaning studies such as ours may become increasingly challenging in the future.

It should be noted that variability of departure in migrant bird species is not solely determined by environmental differences, but also by genetic and physiological differences among individuals (Risely et al. 2015). Migratory behaviours are tuned to external timing cues, such as temperature and this is regulated by polymorphic genes, which could explain consistent among‐individual differences in migratory behaviours (Visser et al. 2010; Lehmann et al. 2012). Individual physiological responses to environmental stressors also vary, which too can explain phenological differences. Birds in better condition with higher fat reserves or greater muscle mass may be better equipped to cope with the physical demands of migration, allowing them to depart earlier or travel longer distances (Hedenström 2008). Exploratory analyses of our data did not identify bird size or mass as significant predictors in explaining phenological variation, but such analyses are better suited for more fine‐scale datasets. Measures like bird mass are highly variable and context‐dependent and are more of a long‐term trait and thus an unreliable indicator of an individual's overall migratory strategy or timing. It is also worth noting that long‐term ringing schemes often lack detailed body condition scores, fat scores or consistent protocols for mass measurements (e.g., before vs. after feeding). This inconsistency limits the reliability of using mass/size as predictors over long time spans.

To better understand the interplay between environment and decision‐making for adaptive migratory behaviour, we need to better evaluate the relative individual costs of an early departure versus a late departure (Ouwehand et al. 2023). A late departure from a non‐breeding site could risk phenological mismatch with conditions at the breeding site, but migrating earlier may incur costs associated with increased risk of depleted resources or food availability or encountering adverse weather conditions (Saino et al. 2017). Fieldfare appeared to show the steepest regression line, arriving in the UK in autumn migration earlier over the years, suggesting the UK experiencing warmer autumns due to climate change may be reducing risks like food scarcity and cold stress. While evidence of the mechanism for this phenological shift is lacking, it is likely due to the birds arriving earlier at their breeding site in the Spring (Scandinavia) for the same reason—warmer temperatures resulting in earlier insect emergence (primary food source in the breeding season; Hogstad et al. 2003).

Abundance of species caught is changing over time: blackbird, chaffinch, greenfinch and linnet have all shown recent stark declines in numbers caught. This is in line with other studies; for example, greenfinches have been declining in Britain since 2006 (Hanmer et al. 2022). Evidence suggests that finch trichomoniasis is a primary driver (Lawson et al. 2012, 2018). It is a recently emerged disease in wild birds caused by the protozoan parasite *Trichomonas gallinae* and was first detected in Britain in 2005. Importantly, Spurn's ringing data show four of the six declining species (which all happen to be finch species) started to decrease in numbers around the same time, suggesting this disease is a probable cause. Revealing declining and increasing abundances of different bird species demonstrates that species assemblages do change over time in response to environmental variation, as species differ in their adaptive potential and response times. This complements UK‐wide trends, as the 2019 BTO Breeding Bird Survey reported 38/117 bird species were showing long‐term increasing trends in population numbers, and 40/117 were in long‐term decline. Additionally, 22 out of 117 bird species surveyed had short‐term increases, and 14 species had short‐term increases (Harris et al. 2020). Furthermore, species can exhibit highly variable regional population trends at the continental scale (Birdlife 2020), which also may depend on differences in habitat quality, productivity, survival, as well as population size, connectivity and losses during migration (Reif 2013; Jiguet et al. 2019; Reif et al. 2023).

Notably, we found that abundance patterns could often be explained by capture effort, which was controlled for in all other analyses. Increased ringing effort results in an increased likelihood of catching new species. This evidences the value behind ringing as a monitoring tool if there are sufficient resources and people to ensure ornithological coverage across the reserve all year round. However, it also serves as a useful cautionary note when seeking to make meaningful interpretations from ringing data. While climatic changes can influence migratory timing, methodological and ecological factors must also be considered when interpreting apparent shifts. Survey effort and timings must be as fixed as possible and only changed in extreme circumstances. While the number of volunteers may vary throughout the year, the number of mist nets remains the same and thus effort should not be impacted. Spurn Bird Observatory also has a Warden who oversees all ringing activity, which minimises changes in observers conducting the surveys that could lead to inconsistencies in data collection. One factor that may contribute to our findings is changes in site habitat conditions. While net rides are actively maintained by the ringing team, the surrounding habitat will change across seasons, and this will influence things like net detectability by birds. However, these changes should be constant across years and have no effect on season‐specific analyses. Large‐scale and long‐term monitoring by volunteer‐led citizen science projects generates larger datasets, but they can be biassed and/or misinterpreted (Legg and Nagy 2005; Jäckel et al. 2021). Given that data collected at multiple locations and spatial scales are essential to detect variation in factors affecting species throughout their range (Díaz et al. 2015; Brlík et al. 2021), citizen science projects have expanded in recent decades for example, eBird, Big Garden Birdwatch. Rigorous data cleaning must be done to ensure the data meets high‐quality checks. This is why the BTO oversees all ringing activity and its data, to ensure there is a regulatory framework in place (BTO 2024a, 2024b). When standardised, ringing data can help us to determine the status of species by considering the distribution and direction of population‐level changes. Moreover, we can then try to identify the causes of these changes and use forecast modelling to predict future consequences (Sæther et al. 2004; Marsh and Trenham 2008).

Going forward, we need to further make use of ringing datasets by analysing individual‐level data. There is a plethora of literature on climate change effects on bird phenology, but only a small portion of these studies is based on individual data, despite this data being key to quantifying the relative importance of plastic versus evolutionary responses (Charmantier and Gienapp 2013). More studies are needed to directly evidence evolutionary responses of bird phenology to current climate change, that is, microevolutionary changes. Rapid improvements in techniques for gathering and analysing individual data offer exciting possibilities that should encourage research activity to fill this knowledge gap, and making use of longitudinal ringing datasets from bird observatories can facilitate this.

## 5 Conclusion

Compared with other animal taxa, birds are well adapted for surviving and reproducing in important and complex habitats. Many bird species have evolved anatomical and physiological adaptations, in addition to life‐history strategies such as timing of reproduction and migration, to facilitate their responses to environmental stressors. Here, we show that varied species have different responses to environmental challenges. The climate at Spurn is changing over time, and some species are able to respond and thus are increasing in number, whereas others are not coping well and are in decline. Given the large diversity of birds at Spurn, inclusive of resident and migratory species, we do not detect any significant changes in species richness in the area. However, species assemblages are indeed changing, and migratory species are changing their migration timings in line with changing environmental conditions. This demonstrates how longitudinal ringing datasets can be used to understand how species are responding to conservation challenges, and importantly, predict future responses and inform conservation for species‐targeted interventions. However, handling ringing data can be challenging and care must be taken when making ecologically meaningful interpretations that could ultimately shape conservation efforts at different hierarchical levels.

## Author Contributions

**Danielle L. Hinchcliffe:** conceptualization (lead), data curation (lead), investigation (lead), project administration (lead), writing – original draft (lead), writing – review and editing (lead). **Patrick Tkaczynski:** formal analysis (lead), investigation (supporting), methodology (equal), writing – review and editing (supporting).

## Disclosure

Statement of inclusion: This study utilises a long‐term citizen science dataset from the British Trust for Ornithology (BTO) ringing scheme, recognising the invaluable contributions of volunteers in ecological research. We acknowledge the importance of inclusive participation in citizen science and the broader scientific community, valuing contributions from individuals of all backgrounds.

## Conflicts of Interest

The authors declare no conflicts of interest.

## Supporting information


**Table S1:** Individual species included in data analysis based on selection criterion described in the methods. Spurn status is taken from Roadhouse (2016). Species in shaded cells appear in all models; the remaining species were included in our analyses of abundance but arrival dates or residence in spring and autumn.
**Figure S1:** Posterior predictive checks for the three climate models. Dark blue lines represent the observed data; the light blue lines represent 100 draws from the posterior.
**Figure S2:** Posterior predictive checks for the phenology and abundance models. Dark blue lines represent the observed data; the light blue lines represent 100 draws from the posterior.
**Figure S3:**. Correlations between yearly averages in Spurn and UK‐wide yearly averages for all climatic variables included in our study (*n* = 25 years).

## Data Availability

All ringing data can be accessed from the DemOn database by ringing‐permit holders licenced by the British Trust of Ornithology (https://app.bto.org/demography/bto/public/login.jsp). Analyses information can be accessed here: https://github.com/ptkaczynski/climate_change_birds.

## References

[ece372317-bib-0001] Bell, F. , J. Ouwehand , C. Both , et al. 2024. “Individuals Departing Non‐Breeding Areas Early Achieve Earlier Breeding and Higher Breeding Success.” Scientific Reports 14, no. 1: 4075. 38374332 10.1038/s41598-024-53575-2 PMC10876959

[ece372317-bib-0002] BirdLife International IUCN Red List . 2020. “Keep Albatrosses Off the Hook.” http://www.birdlife.org.

[ece372317-bib-0003] Border, J. A. , P. H. Boersch‐Supan , J. W. Pearce‐Higgins , et al. 2024. “Spatial Variation in Spring Arrival Patterns of Afro‐Palaearctic Bird Migration Across Europe.” Global Ecology and Biogeography 33: e13850.

[ece372317-bib-0004] Both, C. , and M. E. Visser . 2001. “Adjustment to Climate Change Is Constrained by Arrival Date in a Long‐Distance Migrant Bird.” Nature 411: 296–298. 11357129 10.1038/35077063

[ece372317-bib-0005] Brlík, V. , E. Šilarová , J. Škorpilová , et al. 2021. “Long‐Term and Large‐Scale Multispecies Dataset Tracking Population Changes of Common European Breeding Birds.” Scientific Data 8: 21. 33772033 10.1038/s41597-021-00804-2 PMC7997925

[ece372317-bib-0006] BTO . 2024a. “Bird Ringing Scheme.” https://www.bto.org/our‐science/projects/bird‐ringing‐scheme.

[ece372317-bib-0007] BTO . 2024b. “Demography Online (DeMON) Database.” https://app.bto.org/demography/birdtrends/.

[ece372317-bib-0008] Bürkner, P. 2018. “Advanced Bayesian Multilevel Modeling With the R Package Brms.” R Journal 10, no. 1: 395–411.

[ece372317-bib-0009] Calvert, A. M. , G. Gauthier , and S. Williams . 2021. “Assessing the Biases in Bird Ringing Datasets and Their Impact on Conservation Outcomes.” Avian Conservation and Ecology 16, no. 1: 3.

[ece372317-bib-0010] Charmantier, A. , and P. Gienapp . 2013. “Climate Change and Timing of Avian Breeding and Migration: Evolutionary Versus Plastic Changes.” Evolutionary Applications 7: 15–28. 24454545 10.1111/eva.12126 PMC3894895

[ece372317-bib-0011] Cleland, E. E. , I. Chuine , A. Menzel , H. A. Mooney , and M. D. Schwartz . 2007. “Shifting Plant Phenology in Response to Global Change.” Trends in Ecology & Evolution 22: 357–365. 17478009 10.1016/j.tree.2007.04.003

[ece372317-bib-0012] Cox, J. M. , M. Fitzsimmons , and C. D. Thomas . 2020. “Leveraging Bird Ringing Data to Study Changes in Avian Migration Patterns Over Time.” Ecology Letters 23: 847–857.

[ece372317-bib-0013] Croxton, P. J. , T. H. Sparks , M. Cade , and R. G. Loxton . 2006. “Trends and Temperature Effects in the Arrival of Spring Migrants in Portland (United Kingdom) 1959–2005.” Acta Ornithologica 41: 103–111.

[ece372317-bib-0014] Díaz, M. , J. J. Cuervo , T. Grim , et al. 2015. “Interactive Effects of Fearfulness and Geographical Location on Bird Population Trends.” Behavioral Ecology 26: 716–721.

[ece372317-bib-0015] Dornelas, M. , A. E. Magurran , S. T. Buckland , et al. 2013. “Quantifying Temporal Change in Biodiversity: Challenges and Opportunities.” Proceedings of the Royal Society B: Biological Sciences 280: 1–10. 10.1098/rspb.2012.1931 PMC3574422 23097514

[ece372317-bib-0016] Dunn, P. O. , and D. W. Winkler . 2019. “Changes in Timing of Breeding and Reproductive Success in Birds.” Effects of Climate Change on Birds 9: 113–128.

[ece372317-bib-0017] Dunn, P. O. , D. W. Winkler , A. P. Møller , W. Fiedler , and P. Berthold . 2010. “Effects of Climate Change on Timing of Breeding and Reproductive Success in Birds.” Effects of Climate Change on Birds 11: 17.

[ece372317-bib-0018] Ferger, S. W. , M. Schleuning , A. Hemp , K. M. Howell , and K. Böhning‐Gaese . 2014. “Food Resources and Vegetation Structure Mediate Climatic Effects on Species Richness of Birds.” Global Ecology and Biogeography 23: 541–549.

[ece372317-bib-0019] Fox, J. , and S. Weisberg . 2019. An R Companion to Applied Regression. 3rd ed. Sage. https://www.john‐fox.ca/Companion/.

[ece372317-bib-0020] Gillings, S. 2022. “The Impact of Covid‐19 on the UK Breeding Bird Survey and the Production of Population Trends.” Bird Census News 35: 6–9.

[ece372317-bib-0021] Hanmer, H. J. , A. A. Cunningham , S. K. John , et al. 2022. “Habitat‐Use Influences Severe Disease‐Mediated Population Declines in Two of the Most Common Garden Bird Species in Great Britain.” Scientific Reports 12: 15055. 36064956 10.1038/s41598-022-18880-8 PMC9445085

[ece372317-bib-0022] Harris, S. J. , D. Massimino , D. E. Balmer , et al. 2020. “The Breeding Bird Survey 2019.” In BTO Research Report, vol. 726. British Trust for Ornithology, Thetford.

[ece372317-bib-0023] Hawkins, B. A. , R. Field , H. V. Cornell , et al. 2003. “Energy, Water, and Broad‐Scale Geographic Patterns of Species Richness.” Ecology 84, no. 12: 3105–3117.

[ece372317-bib-0024] Hawkins, B. A. , E. E. Porter , and J. A. F. Diniz‐Filho . 2003. “Productivity and History as Predictors of the Latitudinal Diversity Gradient of Terrestrial Birds.” Ecology 84: 1608–1623.

[ece372317-bib-0025] Hedenström, A. 2008. “Adaptations to Migration in Birds: Behavioural Strategies, Morphology and Scaling Effects.” Philosophical Transactions of the Royal Society, B: Biological Sciences 363: 287–299. 10.1098/rstb.2007.2140 PMC2606751 17638691

[ece372317-bib-0026] Hogstad, O. , V. Selås , and K. Sverre . 2003. “Explaining Annual Fluctuations in Breeding Density of Fieldfares *Turdus Pilaris*–Combined Influences of Factors Operating During Breeding, Migration and Wintering.” Journal of Avian Biology 34, no. 4: 350–354.

[ece372317-bib-0027] Hüppop, O. , and K. H. Hüppop . 2003. “North Atlantic Oscillation and Timing of Spring Migration in Birds.” Proceedings of the Royal Society B: Biological Sciences 270: 233–240. 10.1098/rspb.2002.2236 PMC1691241 12614571

[ece372317-bib-0028] Intergovernmental Panel on Climate Change (IPCC) . 2021. Climate Change 2021: The Physical Science Basis. Cambridge University Press. https://www.ipcc.ch/report/ar6/wg1/.

[ece372317-bib-0029] Jäckel, D. , K. G. Mortega , U. Sturm , U. Brockmeyer , O. Khorramshahi , and S. L. Voigt‐Heucke . 2021. “Opportunities and Limitations: A Comparative Analysis of Citizen Science and Expert Recordings for Bioacoustic Research.” PLoS One 16: e0253763. 34181671 10.1371/journal.pone.0253763 PMC8238189

[ece372317-bib-0030] Jetz, W. , C. Rahbek , and J. W. Lichstein . 2005. “Local and Global Approaches to Spatial Data Analysis in Ecology.” Global Ecology and Biogeography 14: 97–98.

[ece372317-bib-0031] Jiguet, F. , M. Burgess , K. Thorup , et al. 2019. “Desert Crossing Strategies of Migrant Songbirds Vary Between and Within Species.” Scientific Reports 9: 20248. 31882957 10.1038/s41598-019-56677-4 PMC6934701

[ece372317-bib-0032] Langan, J. A. , G. Puggioni , C. A. Oviatt , M. E. Henderson , and J. S. Collie . 2021. “Climate Alters the Migration Phenology of Coastal Marine Species.” Marine Ecology Progress Series 660: 1–18.

[ece372317-bib-0033] Lawson, B. , R. A. Robinson , K. M. Colvile , et al. 2012. “The Emergence and Spread of Finch Trichomonosis in the British Isles.” Philosophical Transactions of the Royal Society, B: Biological Sciences 367: 2852–2863. 10.1098/rstb.2012.0130 PMC3427565 22966140

[ece372317-bib-0034] Lawson, B. , R. A. Robinson , M. P. Toms , K. Risely , S. MacDonald , and A. A. Cunningham . 2018. “Health Hazards to Wild Birds and Risk Factors Associated With Anthropogenic Food Provisioning.” Philosophical Transactions of the Royal Society, B: Biological Sciences 373: 20170091. 10.1098/rstb.2017.0091 PMC5882997 29531146

[ece372317-bib-0035] Legg, C. J. , and L. Nagy . 2005. “Why Most Conservation Monitoring Is, but Need Not Be, a Waste of Time.” Journal of Environmental Management 78: 194–199. 16112339 10.1016/j.jenvman.2005.04.016

[ece372317-bib-0036] Lehikoinen, E. S. , T. H. Sparks , and M. Zalakevicius . 2004. “Arrival and Departure Dates.” In Advances in Ecological Research, vol. 35, 1–31. Elsevier.

[ece372317-bib-0037] Lehmann, M. , K. Spoelstra , M. E. Visser , and B. Helm . 2012. “Effects of Temperature on Circadian Clock and Chronotype: An Experimental Study on a Passerine Bird.” Chronobiology International 29: 1062–1071. 22881370 10.3109/07420528.2012.707159

[ece372317-bib-0038] Marsh, D. M. , and P. C. Trenham . 2008. “Tracking Current Trends in Plant and Animal Population Monitoring.” Conservation Biology 22: 647–655. 18445076 10.1111/j.1523-1739.2008.00927.x

[ece372317-bib-0039] Martin, J. S. , E. Ringen , P. Duda , and V. A. Jaeggi . 2020. “Harsh Environments Promote Alloparental Care Across Human Societies.” Proceedings of the Royal Society B: Biological Sciences 287, no. 1933: 20200758. 10.1098/rspb.2020.0758. PMC7482265 32811302

[ece372317-bib-0040] McElreath, R. 2020. Statistical Rethinking: A Bayesian Course With Examples in R and STAN. CRC Press.

[ece372317-bib-0041] McShane, B. B. , D. Gal , A. Gelman , C. Robert , and J. L. Tackett . 2019. “Abandon Statistical Significance.” American Statistician 73, no. Sup 1: 235–245. 10.1080/00031305.2018.1527253.

[ece372317-bib-0042] Met Office . 2023. “Annual Report and Accounts 2023 to 2024.” https://www.gov.uk/government/publications/met‐office‐annual‐report‐and‐accounts‐2023‐to‐2024.

[ece372317-bib-0043] Met Office , D. Hollis , M. McCarthy , M. Kendon , T. Legg , and I. Simpson . 2021. HadUK‐Grid Gridded Climate Observations on a 5km Grid Over the UK, v1.0.3.0 (1862–2020). NERC EDS Centre for Environmental Data Analysis.

[ece372317-bib-0044] Møller, A. P. , D. Rubolini , and E. Lehikoinen . 2008. “Populations of Migratory Bird Species That Did Not Show a Phenological Response to Climate Change Are Declining.” Proceedings of the National Academy of Sciences of the United States of America 105: 16195–16200. 18849475 10.1073/pnas.0803825105 PMC2571031

[ece372317-bib-0045] Norris, D. R. , J. Schaefer , and G. C. Hays . 2019. “Bird Migration and Conservation: Insights From Long‐Term Ringing Data.” Biological Conservation 238: 108253.

[ece372317-bib-0046] Ouwehand, J. , A. A. Asso , B. Johnston , et al. 2023. “Experimental Food Supplementation at African Wintering Sites Allows for Earlier and Faster Fuelling and Reveals Large Flexibility in Spring Migration Departure in Pied Flycatchers.” Ardea 111, no. 1: 343–370.

[ece372317-bib-0047] R Core Team . 2022. R: A Language and Environment for Statistical Computing. R Foundation for Statistical Computing. https://www.R‐project.org/.

[ece372317-bib-0048] Rahbek, C. , N. J. Gotelli , R. K. Colwell , G. L. Entsminger , T. F. L. V. B. Rangel , and G. R. Graves . 2007. “Predicting Continental‐Scale Patterns of Bird Species Richness With Spatially Explicit Models.” Proceedings of the Royal Society B: Biological Sciences 274: 165–174. 10.1098/rspb.2006.3700 PMC1685854 17148246

[ece372317-bib-0049] Reif, J. 2013. “Long‐Term Trends in Bird Populations: A Review of Patterns and Potential Drivers in North America and Europe.” Acta Ornithologica 48: 1–16.

[ece372317-bib-0050] Reif, J. , J. Koleček , F. Morelli , and Y. Benedetti . 2023. “Population Trends of Ground‐Nesting Birds Indicate Increasing Environmental Impacts From Eastern to Western Europe: Different Patterns for Open‐Habitat and Woodland Species.” Frontiers in Environmental Science 11: 1156360.

[ece372317-bib-0051] Risely, A. , E. Blackburn , and W. Cresswell . 2015. “Patterns in Departure Phenology and Mass Gain on African Non‐Breeding Territories Prior to the Sahara Crossing in a Long‐Distance Migrant.” Ibis 157: 808–822.

[ece372317-bib-0065] Roadhouse, A. 2016. The Birds of Spurn. Spurn Bird Observatory Trust.

[ece372317-bib-0052] Sæther, B. E. , W. J. Sutherland , and S. Engen . 2004. “Climate Influences on Population Dynamics.” Advances in Ecological Research 35: 185–209.

[ece372317-bib-0053] Saino, N. , R. Ambrosini , M. Caprioli , et al. 2017. “Sex‐Dependent Carry‐Over Effects on Timing of Reproduction and Fecundity of a Migratory Bird.” Journal of Animal Ecology 86, no. 2: 239–249. 28000219 10.1111/1365-2656.12625

[ece372317-bib-0054] Schloerke, B. , D. Cook , J. Larmarange , et al. 2024. “GGally: Extension to ‘ggplot2’, R Package Version 2.2.1.” https://github.com/ggobi/ggally. https://ggobi.github.io/ggally/.

[ece372317-bib-0055] Skagen, S. K. , and A. A. Y. Adams . 2012. “Weather Effects on Avian Breeding Performance and Implications of Climate Change.” Ecological Applications 22, no. 4: 1131–1145. 22827123 10.1890/11-0291.1

[ece372317-bib-0056] Socolar, J. B. , P. N. Epanchin , S. R. Beissinger , and M. W. Tingley . 2017. “Phenological Shifts Conserve Thermal Niches in North American Birds and Reshape Expectations for Climate‐Driven Range Shifts.” Proceedings of the National Academy of Sciences 114, no. 49: 12976–12981. 10.1073/pnas.1705897114 PMC5724251 29133415

[ece372317-bib-0057] Sparks, T. H. 1999. “Phenology and the Changing Pattern of Bird Migration in Britain.” International Journal of Biometeorology 42: 134–138.

[ece372317-bib-0058] Sparks, T. H. , K. Huber , R. L. Bland , et al. 2007. “How Consistent Are Trends in Arrival (and Departure) Dates of Migrant Birds in the UK?” Journal of Ornithology 148, no. 4: 503–511.

[ece372317-bib-0059] Stan Development Team . 2021. “RStan: The R Interface to Stan. R Package Version 2.26.24.” https://mc‐stan.org/.

[ece372317-bib-0060] Van Buskirk, J. , R. S. Mulvihill , and R. C. Leberman . 2009. “Complex and Variable Dynamics of Migration Phenology in Eastern North American Songbirds Associated With Climate Change.” Global Change Biology 15: 760–771.

[ece372317-bib-0061] Visser, M. E. , S. P. Caro , K. van Oers , S. V. Schaper , and B. Helm . 2010. “Phenology, Seasonal Timing and Circannual Rhythms: Towards a Unified Framework.” Philosophical Transactions of the Royal Society, B: Biological Sciences 365: 3113–3127. 10.1098/rstb.2010.0111 PMC2981940 20819807

[ece372317-bib-0062] Visser, M. E. , A. J. van Noordwijk , J. M. Tinbergen , and C. M. Lessells . 1998. “Warmer Springs Lead to Mistimed Reproduction in Great Tits (*Parus Major*).” Proceedings of the Royal Society of London, Series B: Biological Sciences 265: 1867–1870.

[ece372317-bib-0063] Waide, R. B. , M. R. Willig , C. F. Steiner , G. Mittelbach , L. Gough , and S. I. Dodson . 1999. “The Relationship Between Productivity and Species Richness.” Annual Review of Ecology and Systematics 30: 257–300.

[ece372317-bib-0064] Wiens, J. J. , and J. Zelinka . 2024. “How Many Species Will Earth Lose to Climate Change?” Global Change Biology 30, no. 1: e17125. 38273487 10.1111/gcb.17125

