# Supplementary material for: Shifting Seasons: Long‐Term Insights Into Climate Change Effects on Bird Phenology From Ringing Data
Source: Ecol Evol. 2025 Oct 10;15(10):e72317. doi: 10.1002/ece3.72317 (PMC12513722; doi:10.1002/ece3.72317)
Supplement: Supplementary file 1 — Table S1: Individual species included in data analysis based on selection criterion described in the methods. Spurn status is taken from Roadhouse (2016). Species in shaded cells appear in all models; the remaining species were included in our analyses of abundance but arrival dates or residence in spring and autumn. Figure S1: Posterior predictive checks for the three climate models. Dark blue lines represent the observed data; the light blue lines represent 100 draws from the posterior. Figure S2: Posterior predictive checks for the phenology and abundance models. Dark blue lines represent the observed data; the light blue lines represent 100 draws from the posterior. Figure S3:. Correlations between yearly averages in Spurn and UK‐wide yearly averages for all climatic variables included in our study (n = 25 years). [file ECE3-15-e72317-s001.docx]

**Supplementary material**

**Table S1.** Individual species included in data analysis based on selection criterion described in the methods. Spurn status is taken from Roadhouse (2016). Species in shaded cells appear in all models; the remaining species were included in our analyses of abundance but arrival dates or residence in spring and autumn.

| **Common name** | **Scientific name** | **Order** | **Spurn Status** |
| --- | --- | --- | --- |
| Blackbird | *Turdus merula* | Passeriformes | Resident and migratory |
| Blackcap | *Sylvia atricapilla* | Passeriformes | Resident and migratory |
| Brambling | *Fringilla montifringilla* | Passeriformes | Migratory |
| Chaffinch | *Fringilla coelebs* | Passeriformes | Resident and migratory |
| Chiffchaff | *Phylloscopus collybita* | Passeriformes | Migratory |
| Dunlin | *Calidris alpina* | Charadriiformes | Migratory |
| Dunnock | *Prunella modularis* | Passeriformes | Resident and migratory |
| Fieldfare | *Turdus pilaris* | Passeriformes | Migratory |
| Goldcrest | *Regulus regulus* | Passeriformes | Resident and migratory |
| Goldfinch | *Carduelis carduelis* | Passeriformes | Resident and migratory |
| Greenfinch | *Chloris chloris* | Passeriformes | Resident and migratory |
| Knot | *Calidris canutus* | Charadriiformes | Migratory |
| Linnet | *Linaria cannabina* | Passeriformes | Migratory |
| Redshank | *Tringa totanus* | Charadriiformes | Migratory |
| Redstart | *Phoenicurus phoenicurus* | Passeriformes | Migratory |
| Redwing | *Turdus lilacus* | Passeriformes | Migratory |
| Robin | *Erithacus rubecula* | Passeriformes | Resident and migratory |
| Siskin | *Spinus spinus* | Passeriformes | Resident and migratory |
| Starling | *Sturnus vulgaris* | Passeriformes | Resident and migratory |
| Swallow | *Hirundo rustica* | Passeriformes | Resident and migratory |
| Swift | *Apus apus* | Passeriformes | Migratory |
| Waxwing | *Bombycilla garrulus* | Passeriformes | Migratory |
| Wheatear | *Oenanthe oenanthe* | Passeriformes | Migratory |
| Whitethroat | *Sylvia communis* | Passeriformes | Resident and migratory |
| Wren | *Troglodytes troglodytes* | Passeriformes | Resident and migratory |

*Common and lesser redpoll have since been merged into a single species


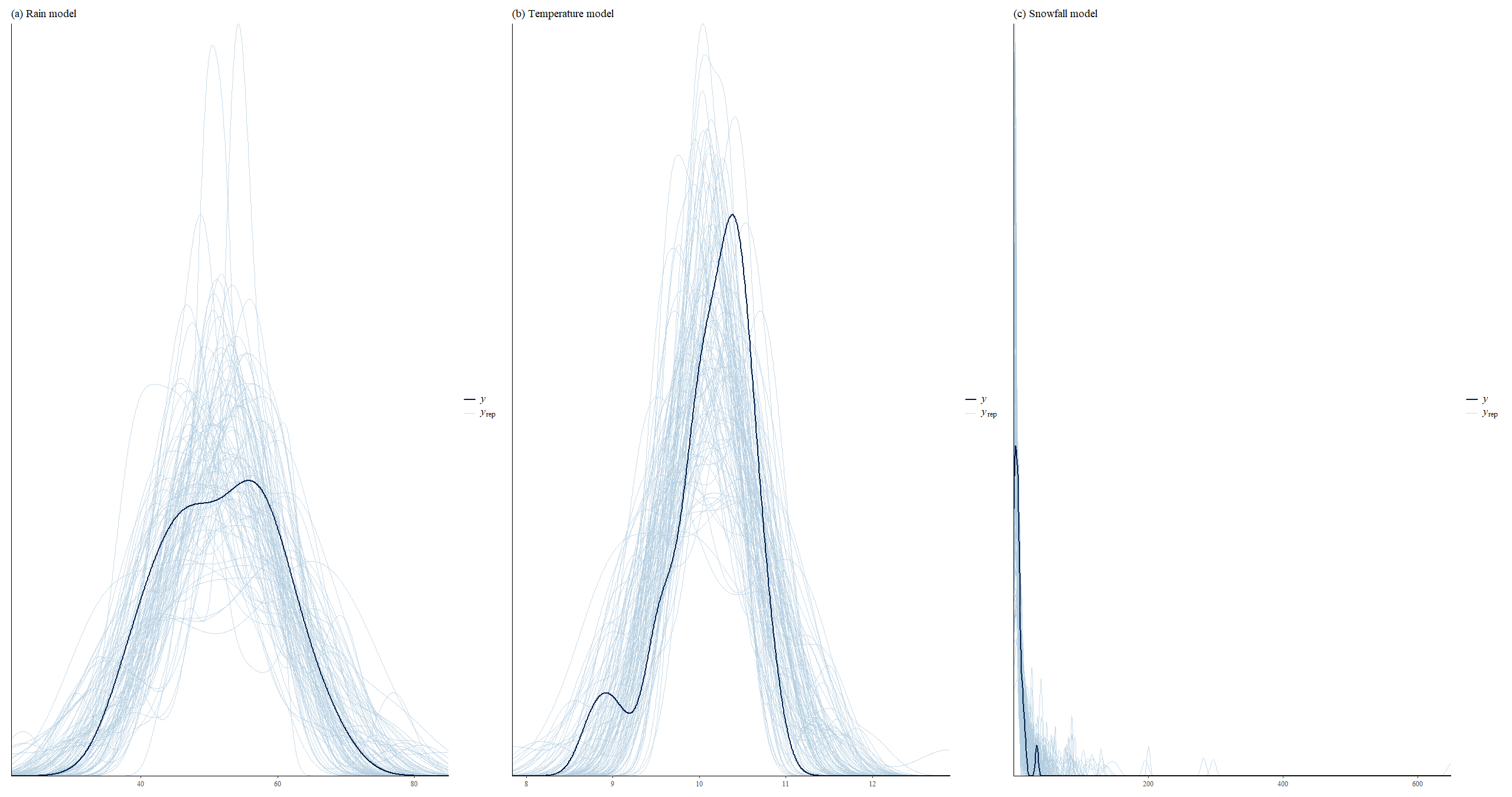


**Figure S1.** Posterior predictive checks for the three climate models. Dark blue lines represent the observed data; the light blue lines represent 100 draws from the posterior.


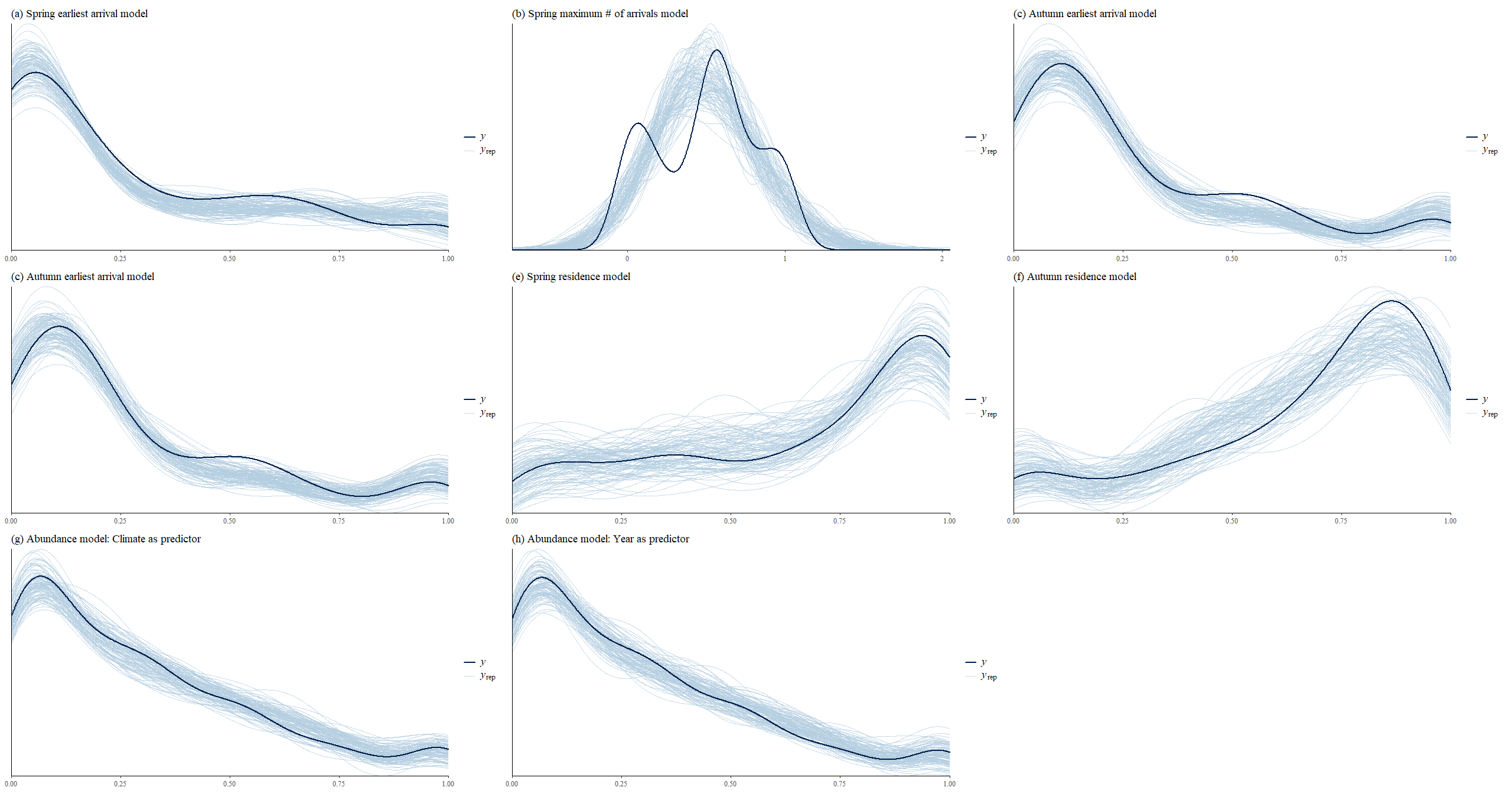


**Figure S2.** Posterior predictive checks for the phenology and abundance models. Dark blue lines represent the observed data; the light blue lines represent 100 draws from the posterior.


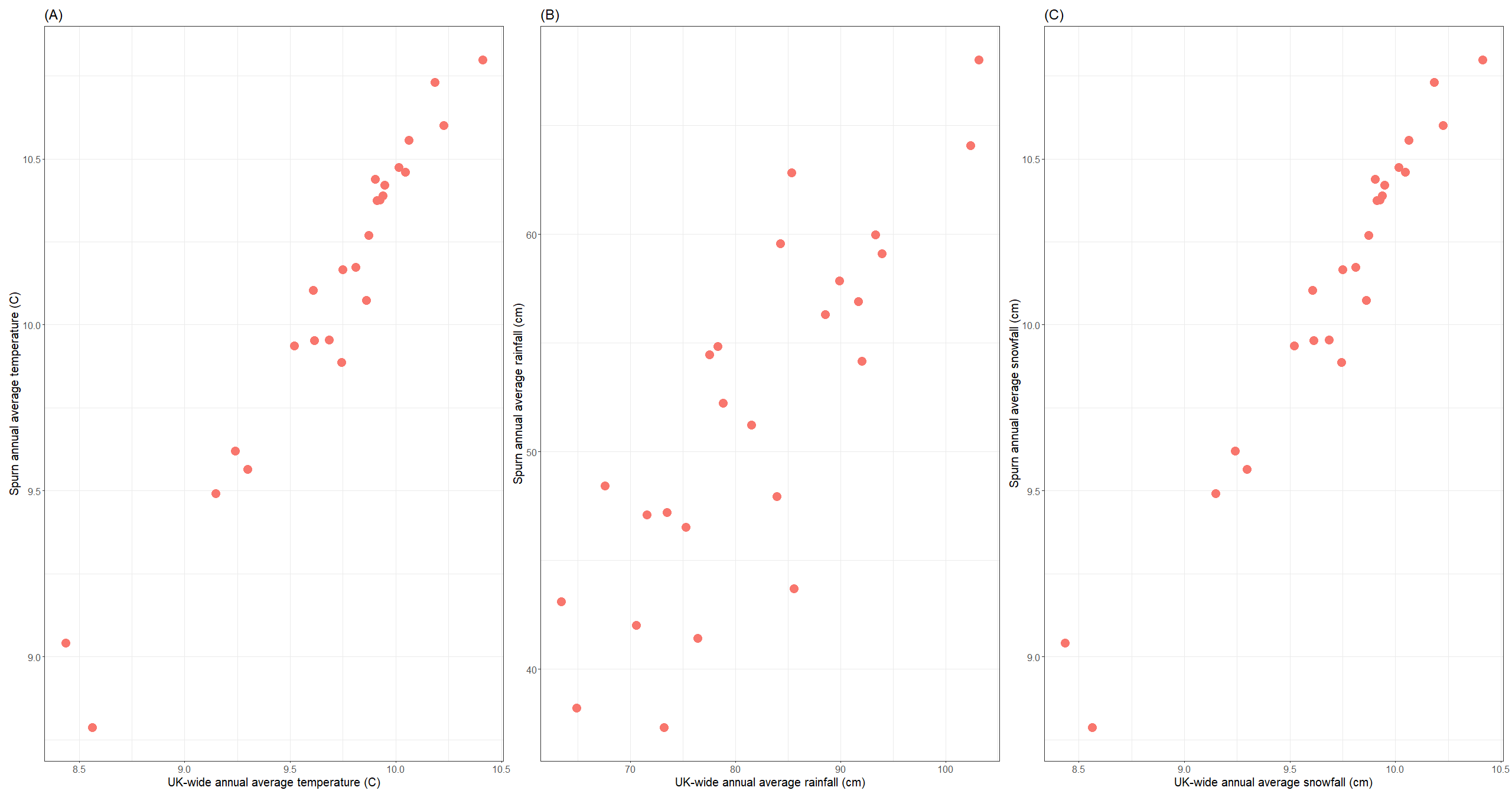


**Figure S3.** Correlations between yearly averages in Spurn and UK-wide yearly averages for all climatic variables included in our study (n=25 years).
